# Defensive medicine through the lens of the managerial perspective: a literature review

**DOI:** 10.1186/s12913-023-10089-3

**Published:** 2023-10-17

**Authors:** Gianfranco Pischedda, Ludovico Marinò, Katia Corsi

**Affiliations:** Department of Economics and Business, University of Sassari, Via Muroni, 25, 07100 Sassari, Italy

**Keywords:** Defensive medicine, Healthcare sector, Management issue, Managerial practices, Literature review

## Abstract

**Purpose:**

Several studies have been carried out on defensive medicine, but research from the managerial viewpoint is still scarce. Therefore, the aim of the present study is to conduct a literature review to better understand defensive medicine from a managerial perspective.

**Design/methodology/approach:**

A literature review was conducted of studies focusing on the organisational (meso) level of healthcare providers and managerial practices. A final sample of 28 studies was processed.

**Findings:**

Defensive medicine has mainly been studied in the USA, and scholars have principally used quantitative surveys. High-risk specialities have been a critical field of investigation, and a large portion of the papers are published in journals that cover medicine, health policy, education and law fields. The analysis showed that operations and the organisation of staffing were the most discussed managerial practices. No study considered planning and budgeting aspects.

**Originality/value:**

The review confirmed that the managerial aspect of defensive medicine has not been fully addressed. Stimulated by this gap, this study analyses the managerial background of the defensive medicine phenomenon and shows which managerial practices have been most analysed. This paper also contributes to developing the literature on defensive medicine from the managerial side. Areas for future research include qualitative studies to investigate the behaviour of managers of healthcare companies to give a different perspective on defensive medicine and organisations’ decision-making.

**Research limitations/implications:**

Some important publications might have been missed in this work because of the choice of only two databases. A further limit could be imposed by the use of the English language as an inclusion criterion.

## Introduction

Defensive medicine can be defined as the avoidance of specific high-risk procedures or patients or the ordering of unnecessary procedures, tests, or visits assessment [64]. For this reason, defensive medicine represents a cancer in the health system, as it does not allow patients to receive high-quality care in line with physicians’ moral, legal, and ethical duties [10]. Additionally, defensive medicine practices aimed at minimising malpractice liability may negatively affect the system by lowering the quality of care and increasing costs. Studies conducted by authors from Harvard University and the University of Melbourne estimated that defensive medicine costs America $45.6 billion annually (in 2008 dollars), accounting for more than 80% of the $55.6 billion total yearly cost of the medical liability system. Michelle Mello, one of the authors from the Harvard School of Public Health, estimates defensive medicine costs both for hospitals ($38.8 billion) and for physicians ($6.8 billion) [19]. In Europe, the number of litigations for medical malpractice has increased significantly over the last decade (from 50% in Britain, Scandinavia, the Baltic countries and Eastern Europe to more than 200% in the Mediterranean Area countries) [61]. A study published in 2015 by the Italian Ministry of Health estimated that defensive medicine costs represent the first category of healthcare waste, representing 0.75% of the Italian GDP. Nevertheless, accurately determining the cost of defensive medicine can be complex, as its computation occasionally approximates the entire healthcare wastage, of which defensive practices represent just one facet [38]. Nonetheless, more recent research has proposed that the comprehensive expense associated with defensive practices spans from $46 billion to $300 billion, with most estimates falling within the $50 to $65 billion bracket [15, 39, 46].

The critical thing is that this situation seems destined to worsen especially following the Covid-19 pandemic. Indeed, the limited treatment algorithms and the absence of guidelines for managing the disease, caused by the extraordinary nature of the event, face the highest malpractice claims, particularly for specialists such as surgeons, obstetricians, and gynaecologists with a consequent potential increase of defensive medicine [18, 49].

Although multiple contributions have been made to the study of defensive medicine in the literature, it is difficult to find a complete and systemic vision of the problem due to its multifaceted issue that concerns medical, economic, and legal considerations and the beliefs and values of a society. In medical contributions, on the one hand, the topic is often discussed within individual medical specialities, particularly regarding those with a high risk of litigation, such as emergency medicine, general surgery, orthopaedic surgery, neurosurgery, obstetrics/gynaecology, and radiology [25, 34]. On the other hand, some contributions focus on physicians, highlighting the different approaches they could take toward their patients or defensive medicine’s emotional and psychological impact shield [12].

The economic and business contributions, instead, emphasise the phenomenon’s implications for the whole health system, especially in terms of extent and costs [74].

Many studies on defensive medicine have aimed to grasp the relevance of defensive medicine and its consequent problems through interviews with physicians working in specific contexts. Although this is the primary way to identify defensive medicine behaviours, it is not without limitations: the defensive behaviours that emerge are only those self-declared by physicians and are therefore affected by numerous subjective elements. For this reason, we still lack a full understanding of the defensive medicine problem, and further clarification has been requested. This point is stressed by a recent literature review that concluded that defensive medicine is confusing from several points of view, causing problems for policymakers, practitioners and the management of healthcare companies when they try to find means to reduce the phenomenon [51].

The present work aims to respond to this research call, addressing defensive medicine by analysing the phenomenon from a managerial perspective. To the best of the researcher’s knowledge, the few literature reviews on this issue pertain to the medical field and patient safety [3] and deal only marginally with its managerial impacts and possible available solutions. Awareness of the phenomenon at an organisational level, including through the implementation of managerial practices and tools able to control and limit defensive medicine and negligent behaviours, is, in fact, important to make health systems more effective and efficient as well as safer. In addition, observing the defensive medicine considering managerial practice could give more unbiased results, not strictly linked to the subjectivity of interviewees.

Thus, inspired by the identified gap, the current work attempts to offer a different perspective on the phenomenon to offer further development possibilities for more targeted research.

In order to achieve that goal, a literature review is performed. This approach examines the features of available knowledge before determining how it can be improved [53]. Only documents at the organisational level (meso) will be considered to keep defensive medicine to a managerial overview, following the classification proposed by Ries & Jansen [26]. In addition, to ensure analytical rigour, only literature on managerial practices will be considered [35, 52, 65, 80]. The paper is organised as follows. The following section presents the theory focused on managerial practices. The third section outlines the methodology, followed by the results. Discussions, future developments, limits of the present research and conclusions are given in the last section.

## Management, managerial practices, and defensive medicine

During the last 30 years, public organisations’ landscapes have changed following globalisation, which has favoured market orientation and the adoption of managerial values, logic, and practices to improve public organisation performances. Notably, according to the new public management (NPM) orientation, the correct use of managerial practices/tools can lead to higher performance and higher service quality by simultaneously reducing citizens’ dissatisfaction [50, 56, 81]. In this new context, managerial practices derived from the private sector have been increasingly adopted by public organisations, including in the healthcare sector – see, for example, Buelow et al. [33], Ezza [67], and Johansen & Zhu [24]– for a simple reason: the adoption of correct managerial practices will guarantee greater chances of growth, success, and survival [65].

A profound performance heterogeneity between organisations – be they public or private – characterises the world economic system. These differences depend on geographical characteristics (not all areas of the world are equal) and sectorial features (organisations and firms that operate in the same sector present a profound variability of results). In this sense, it is possible to assert that these differences can also be traced to the quality of management. Indeed, performance can be affected by both unpredictable factors and more objective dynamics that are manageable through managerial actions. Considering the latter, a key role is played by the (in)correct adoption of managerial practices.

In defining managerial practices, great relevance is given to the works of Bloom and Van Reenen, who contributed enormously to our understanding of the role of such practices in guiding organisational actions and performances [77]. The relevance of Bloom and Van Reenen’s work is strictly connected to the definition of a model able to identify bad and good managerial practices [52]. Such practices are classified into four areas: operations (service delivery standardisation, formalisation, and centralisation of procedures),monitoring (ongoing improvement, performance measurement, performance control),targets (type of objectives adopted, transparency of goals, consistency of objectives including those of the organisation as a whole); and incentives (individual performance assessment, support practices for employee activities).

Since management is a complicated set of processes, technologies, and people, the current study also deals with other features that are considered to be among the most critical aspects of management: planning and budgeting (planning and scheduling of activities; the adoption of performance objectives; the presence of steps for achieving needed results); organising and staffing (adequate use of human resources; delegating responsibility and authority for carrying out the targets; providing policies and procedures to help guide people; creating methods or systems to monitor the implementation of targets and people); and controlling and problem-solving (monitoring results; identifying deviations and organising to solve the problems) [80].

To date, the managerial approach has been applied to defensive medicine only to a limited extent. Defensive medicine – since it identifies a divergence from the healthy and responsible medical practice [45] – has been mainly analysed in terms of its negative impacts on services, the reduction of the quality of treatment/outcome (e.g., lack of access to adequate care, expansion of waiting lists, etc.), and the increase in healthcare-related costs (waste of resources, mismanagement of hospital utilities, etc.) [34, 45]. In particular, recent studies have attempted to discuss the amount of defensive medicine costs [37], the impact of insurance premiums on the healthcare system [5, 19], and the impact of tort reforms on healthcare costs and defensive medicine [9, 63]. However, to the best of the researcher’s knowledge, few works have addressed the managerial side of this practice and the possible solutions available.

Nevertheless, improvements to both patient safety in the care path and the efficiency of a national health system must deal with a modern managerial and organisational culture capable of avoiding and managing errors. The inability to manage error is one of the major causes of defensive medicine [16], Miziara & Miziara,). Ensuring this awareness is present at the organisation (meso) level and planning the activities to be transferred to the level of the individual physician (micro) – who works directly with the patient – leads to safer care process management. A (better) consideration of the managerial practices selected in the current study could provide valuable information to frame the phenomenon from a different and more upstream perspective. To solve problems, it is often necessary to tackle the issue upstream. It is for this reason that the reduction of defensive medicine also involves an organisation being called to consider better the practices proposed by Bloom & Van Reenen [52] and Kotter [80], and specifically:

- Operations that are critical to improving the organisational structure of healthcare organisations and their production cycle. Adopting the resources better also favours an overall improvement in the performance of healthcare organisations in terms of effectiveness, efficiency, and patient satisfaction. Furthermore, the use of procedures and checklists and an accurate standardisation of the activities favour the development and diffusion of repeatable knowledge in specific procedures, reducing errors and avoidable risk elements.
- Organising staffing and incentives, which are among the most critical functions in healthcare organizations [1]. The management of a healthcare organisation must be able to coordinate the various existing professional skills (health and non-healthcare) and activate incentive mechanisms to guide individuals' behaviour towards achieving individual and corporate objectives respecting patients' needs. Identifying practices capable of educating residents and doctors about risk management, supporting doctors, and directing them in their activity is important to ensure and increase business results as well as the quality of the service.
- Planning, budgeting, and setting out targets are among NPM pillars [20, 50, 56, 81]. These practices allow organisations to embark on a righteous path that moves from long-term to short-term planning and enable them to evaluate the causes that led to a specific deviation from the forecast events. In this case, it is essential to develop management tools and well-structured risk and compliance programs to evaluate and classify the nature of errors, to encourage the communication of errors between physicians and programming and control officers and find ways to reduce their recurrence by implementing corrective behaviours that will limit/nullify its repetition in the future.
- Monitoring, controlling, and problem-solving are fundamental practices for allowing a continuous improvement of activities. A clear and structured path for defining problems will enable organizations to circumscribe, tackle, and resolve errors, positively affecting company results. Controlling and monitoring errors is essential to learn from them and implement mechanisms to correct mistakes instead of ignoring them.

All the previous managerial practices, combined at all organisational levels, can improve the performance of organisations [67], as well as patient care and safety, and ensure protection from unnecessary litigation (and costs).

## Methodology

### Study design

This systematic review aims at offering a different overview of the research that investigated the defensive medicine phenomenon from a managerial perspective. This study starts with exploring certain descriptive characteristics of prior research: 1. Year of publication, citation by year and most cited paper; 2. Country of the study; 3. Areas of medical practices and data collection methods; 4. Authors’ affiliation, publishing activities of journals and rankings. Subsequently, the managerial practices related to defensive medicine are examined.

The selection criteria used and the analyses that led to the final sample were developed through discussions between the authors. The various analyses aimed at providing a picture of the current body of research on defensive medicine to fulfil the purpose of the present study and any discrepancies that emerged during the selection of articles were resolved through further discussion and consensus.

### Paper inclusion criteria and data source

**Fig. 1:**
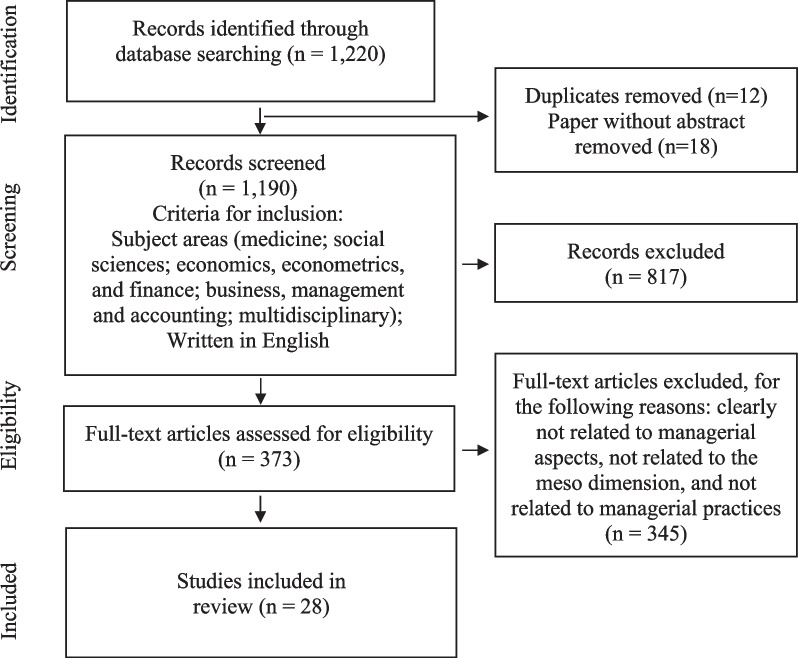
Literature search flow diagram (PRISMA)

The papers considered in this review are those published in English between January 2011 and June 2022 and reporting on studies investigating defensive practices at the organisation (meso) level. Papers were ineligible if they focused on the macro (system) or micro (practitioner) levels precisely because of the authors’ desire to give defensive medicine a managerial overview. The Preferred Reporting Items for Systematic Reviews and Meta-Analyses (PRISMA) diagram (Fig. 1) was used to select the literature on defensive medicine for inclusion in the analysis [70]. The first step involved a search of two major databases: Web of Science (WoS) and Scopus. WoS is the most used database for bibliometric analyses in management and organisation,Scopus is characterised by its more extensive coverage and is thus helpful for mapping fields that are not widely covered by the WoS [28, 30]. The year 2011 was chosen as the starting point for this research because the interest in low-value treatments, and indirectly, defensive medicine, had surged due to the launch of various initiatives started in that period. These initiatives included the Too Much Medicine program by the British Medical Journal in the UK and Choosing Wisely in the USA. These programs brought attention to low-value and unnecessary care, highlighting multiple contributing factors, including the fear of legal risks and defensive medicine [79].

While primarily focused on addressing low-value treatments, these initiatives indirectly combat defensive medicine [38, 41] by promoting the “less is more” approach [58, 72]. Defensive medicine is identified as one of the primary drivers of healthcare overuse [6, 14, 27, 68]. Furthermore, the definition of defensive medicine accepted in this study encompasses ordering unnecessary procedures, tests, or evaluation visits [64], which constitutes overuse.

### Identification phase

In the identification phase, performed in July 2022, the first step was to select articles whose title, keywords, or abstract featured the term ‘defensive medicine’, ‘defensive practice’, or ‘medical malpractice’, published between January 2011 and June 2022 (*n* = 1,220). These words were preferred since the inclusion of managerial terms, such as "costs", "effectiveness", and "efficiency", within the title, keywords, or abstract did not produce significant results. The results were thus refined in the subsequent phases.

### Screening phase

Once duplicates and papers without abstracts have been eliminated, the search was refined to narrow the concept of defensive medicine on management issues. Specifically, the research encompassed the following areas: medical (the most comprehensive domain); social sciences; economics, econometrics and finance; business, management and accounting; and multidisciplinary (*n* = 373). Other areas, such as computer science, psychology, agricultural and biological science, material sciences, were not considered because they were not in line with the main target of the review.

### Eligibility and inclusion criteria

In the eligibility phase, the authors screened titles and abstracts to exclude search results clearly not related to managerial aspects (i.e., not directly connected to organizing, overseeing, or administering healthcare services or facilities such as 1. human resources planning and management, 2. patient care management, 3. healthcare facility management etc.). This further filter was needed because the analysis aimed to identify the impact of defensive medicine and its relevance to the business and management literature. Of 373 articles selected, those not related to the organizational level (meso^1^) and those that did not include any references to managerial practices^2^ were also excluded. As a result, 28 articles were included for the final review.

## Results

In this section, the results of the analysis of defensive medicine are provided for all 28 documents.

### Year of publication, citations by year, and most-cited papers

**Fig. 2:**
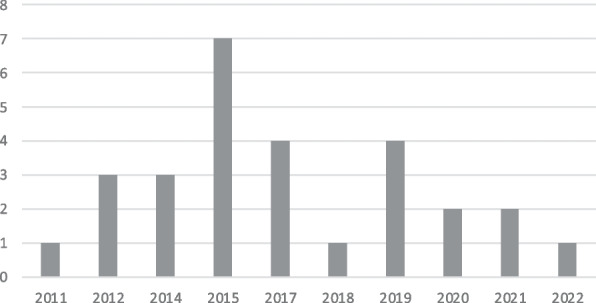
Year of publication

Figure 2 shows the frequency count of publications between 2011 and June 2022, revealing that the most productive year was 2015, with seven publications, followed by 2017 and 2019, with four publications each.

**Fig. 3:**
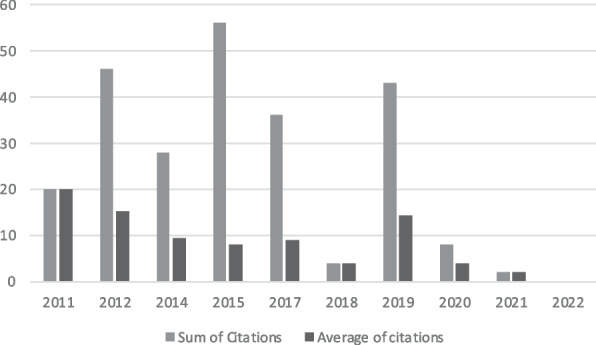
Sum of and average citations per year

The year which saw the highest number of total citations was 2015 (56), followed by 2012 (46) and 2019 (43). Contrariwise, the lowest numbers of citations were recorded (not considering 2022 due to the short time that has passed since publication) in 2021 (2) and 2018 (4). However, as Fig. 3 shows, the average number of citations per year was calculated to appreciate the more representative impact of papers published in a specific year. The most representative year is 2011, thanks to a paper that counts 20 citations and the fact that only one paper was published [8], followed by 2012 (46 citations and three papers published), and 2019 (43 citations and four papers published).

The analysis of the number of citations aims to give a comprehensive framework of papers published in a specific field [11]. This analysis reveals that Ozeke et al. [76] were the most cited (35), followed by OʼLeary et al. [78] and Avraham [8], respectively, with 25 and 20 citations. In their work, Ozeke et al. addressed the problem of secondary victims in healthcare, these being healthcare providers involved in a medical error and/or patient-related injury, who were victimised in the sense of being traumatised by the event. O’Leary et al. assessed medical students’ and residents’ experiences of defensive medicine, stating that medical trainees reported frequently encountering defensive medicine practices and were often taught to consider malpractice liability during clinical decision-making.

Lastly, Avraham addressed the problem of medical errors and overtreatment in the USA by examining the warped incentives that underlie the US system. The tort system, lack of expertise, and slowness to adapt cannot overcome cognitive biases to solve problems adequately. According to the author, moreover, although clinical guidelines could solve these problems, they do not work because they do not operate within a framework of incentives which can appropriately address the disincentives and inefficiencies within the US healthcare system. Lastly, the author proposed a private regulation regime as a potential solution to align all the players’ incentives with society’s interests.

### Authors’ affiliation, publishing activities of journals and rankings

Based on the SCImago Journal Rank (SJR) – which is a measure of the scientific influence of academic journals – most of the selected papers are in journals classified in the medicine research field, followed by health policy, education and law. It also seems appropriate to underline the predominance of the authors’ affiliations within the medical field. In contrast, economics, law and management are less represented and appear three times for economics and law fields and once for management.

Most of academic journals (61%) are in Q1, while the rest in Q2 (25%) and Q3 (7%). Two of the analysed documents are not ranked.

The most prolific journals are BMC Medical Education, BMC Medical Ethics, Academic Medicine and Journal of Forensic and Legal Medicine (two papers each).

Finally, the Academic Journal Guide (AJG) – which provides details on a wide range of journals in which business and management academics may seek to publish their research – was used to assess whether the selected papers were published in a journal that stretches across business and management fields. It is worth noting that there is only one paper, shown in bolt in Table 1.

**Table 1:** Publishing activity of journals and rankings

Publishing activity	Journal	SJR	Coverage
2	BMC Medical Education	Q1	Education (Q1); Medicine (miscellaneous) (Q2)
2	BMC Medical Ethics	Q1	Health Policy (Q1); Health (social science) (Q1); Issues, Ethics and Legal Aspects (Q1)
2	Academic Medicine	Q1	Education (Q1); Medicine (miscellaneous) (Q1)
2	Journal of Forensic and Legal Medicine	Q1	Law (Q1); Pathology and Forensic Medicine (Q2); Medicine (miscellaneous) (Q3)
1	American Journal of Speech-Language Pathology	Q1	Developmental and Educational Psychology (Q1); Linguistics and Language (Q1); Medicine (miscellaneous) (Q1); Otorhinolaryngology (Q1); Speech and Hearing (Q1)
1	Bioethics	Q1	Philosophy (Q1); Health Policy (Q2); Health (social science) (Q2)
1	BMJ Open	Q1	Medicine (miscellaneous) (Q1)
**1**	**European Journal of Health Economics**	**Q1**	**Economics, Econometrics and Finance (miscellaneous) (Q1); Health Policy (Q1)**
1	International Journal of Environmental Research and Public Health	Q1	Health, Toxicology and Mutagenesis (Q1); Pollution (Q2); Public Health, Environmental and Occupational Health (Q2)
1	Journal of Community Health	Q1	Health (social science) (Q1); Public Health, Environmental and Occupational Health (Q1)
1	Journal of Surgical Research	Q1	Surgery (Q1)
1	Perspectives on Medical Education	Q1	Education (Q1); Medicine (miscellaneous) (Q1)
1	Sociology of Health and Illness	Q1	Health Policy (Q1); Health (social science) (Q1); Public Health, Environmental and Occupational Health (Q1)
1	Advances in Medical Education and Practice	Q2	Education (Q2)
1	Health (United Kingdom)	Q2	Health (social science) (Q2)
1	Health Economics, Policy and Law	Q2	Health Policy (Q2)
1	Journal of Legal Medicine	Q2	Law (Q2); Medicine (miscellaneous) (Q3)
1	Medical Law International	Q2	Law (Q2); Medicine (miscellaneous) (Q4)
1	Medical Oncology	Q2	Hematology (Q2); Medicine (miscellaneous) (Q2); Oncology (Q2); Cancer Research (Q3)
1	Medicine, Health Care and Philosophy	Q2	Education (Q2); Health (social science) (Q2); Health Policy (Q3)
1	American Journal of Law and Medicine	Q3	Law (Q3); Health (social science) (Q4); Medicine (miscellaneous) (Q4)
1	Journal of law and medicine	Q3	Law (Q3); Health Policy (Q4); Issues, Ethics and Legal Aspects (Q4); Medicine (miscellaneous) (Q4)

### Country of study

Defensive medicine seems to be discussed worldwide, but with different productivity levels. To determine the impact of different countries, analyses considering the most productive country(ies) by volume and average article citation per country were carried out.

Based on the first analysis, most of the papers analysed in the present research were conducted in a single country, while two documents reported examples or data in more than one country to determine comparative analyses and give an international perspective on defensive medicine and practices [57, 83]. Most of the research was conducted in the USA (*n* = 7), followed by Australia, Germany, Japan, and the UK, with two publications each. It is important to note that the multi-country studies also involved data collected from the USA, increasing the proportion of papers with US data.

**Fig. 4:**
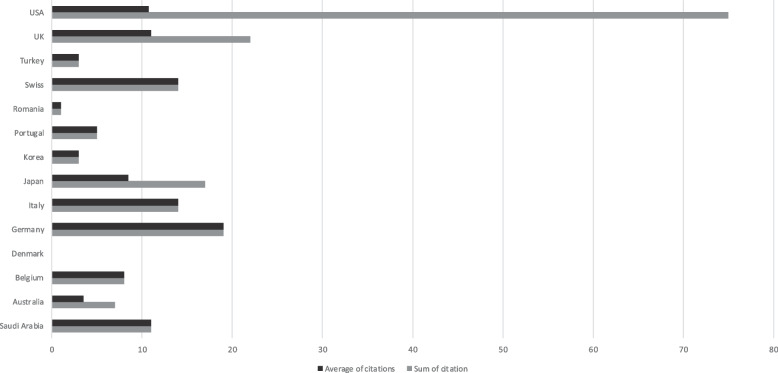
Total and average article citations per country

Total and average article citations per country were calculated to determine the impact and influence of a single country within the research community. As Fig. 4 shows, although the USA also presented the highest number of citations (75), followed by the UK (22), Germany (19), Japan (17), and Italy and Switzerland (14), higher than average numbers of article citations were observed for Germany, Italy, and Switzerland. This result indicates that there is no correspondence between the number of articles published in each country and the average citations per country. The greater diffusion of research themes in a country denotes an amplified interest, which (probably) feeds on itself but does not mean that the works are ever perceived as of higher quality.

### Areas of medical practice and data collection methods

The studies examined defensive medicine in several fields of medicine, sometimes reporting single or mixed examples of clinician fields. Starting from the single examples, obstetrics and/or gynaecology is the most studied field, being addressed by four papers [4, 29, 36, 43], followed by surgery (three papers) [29, 40, 73]. Residual studies consider other fields, such as dentistry [54], upper respiratory tract infections [23], oncology [17], and primary care [7]. This residual category includes a study that considers physicians as second victims of medical errors [76]. Only one work compares fields, namely the study carried out by Sartwelle et al. [29], which presents a comparison of obstetrics, gynaecology, internal medicine, and surgery.

However, most of the documents reporting on defensive medicine consider a variety of medical specialities without pinpointing them precisely (11 papers). For example, Bourne et al. [21] invited British Medical Association (BMA) members to complete an online survey, while Lee [55] conducted an online survey with 79,022 doctors who are members of the Korea Medical Association. Other authors’ analyses of medical specialities also considered the connection between defensive medicine and the problems of communication/sharing information [2, 59]. Finally, four articles reported on studies involving medical students or participants in training programmes (residents) or how the training itself should be carried out to avoid defensive practices [31, 62, 75, 82].

As regards data collection methods, surveys, mainly cross-sectional, were the most common method (*n* = 9). Seven documents reported qualitative data collected via semi-structured interviews (see, for example, Lombardo et al., [57], Mankaka et al., [62] or focus groups involving general practitioners [7]. Two publications provided a qualitative analysis using a case study methodology [47, 48]. In particular, the work of Horner et al. [47] consists of a hypothetical case of a non-compliant individual under the care of an interdisciplinary neurorehabilitation team to illuminate the ethical and legal features of the patient-practitioner relationship. Finally, several papers address the causes and determinants of and potential solutions to defensive medicine in more theoretical terms (e.g. [36, 60, 83]). It is interesting to note that none of the surveys in the studies reviewed, whether qualitative or quantitative, considers managerial/administrative positions or figures. Rather, the phenomenon is always framed from a medical perspective.

### Managerial practices and defensive medicine

In analysing the defensive medicine phenomenon and considering managerial practices as classified in the first part of this work, the most considered practices are operations (16 times) and organising and staffing (15 times), followed by controlling and problem-solving and monitoring (five times), targets (four times), and incentives (three times). No work seems to address planning and budgeting practices. Finally, only one work (i.e. [82] seems to embrace all practices since it explains how activities and outcomes can be enhanced. The improvement of activities, the monitoring of activities, and education towards responsible training are critical elements in cost-effectiveness and, combined with the improvement of quality, are linked to defensive medicine practices and their management/reduction.

Table 2 summarises the works selected and the corresponding managerial practices identified. The selected works are arranged in chronological order, while the practices are presented according to their relevance.

**Table 2:** The works selected and the managerial practices identified

Title	Author(s)	Year	Operations	Organizing & staffing	Controlling & problem solving	Monitoring	Targets	Incentives	Planning & budgeting
Clinical Practice Guidelines: The Warped Incentives in the U.S. Healthcare System	Avraham, R	2011	x		x				
Malpractice liability, technology choice and negative defensive medicine	Feess, E	2012	x						
Opportunities for Cost Reduction of Medical Care: Part 3	Malach, M., & Baumol, W. J	2012		x	x				
Medical students’ and residents’ clinical and educational experiences with defensive medicine	O'Leary, K. J., Choi, J., Watson, K., & Williams, M. V	2012		x				x	
“Innovation” Institutes in Academic Health Centers: Enhancing Value Through Leadership, Education, Engagement, and Scholarship	Pines, J. M., Farmer, S. A., & Akman, J. S	2014	x			x	x	x	
Female residents experiencing medical errors in general internal medicine: a qualitative study	Mankaka, C. O., Waeber, G., & Gachoud, D	2014		x		x	x		
Lessons from a decade of technical-scientific opinions in obstetrical litigation	Domingues, A. P., Moura, P., & Vieira, D. N…	2014		x	x				
A comparison of medical litigation filed against obstetrics and gynecology, internal medicine, and surgery departments	Hamasaki, T., Hagihara, A	2015	x						
Home Blood Pressure Monitoring – One Step Towards Reducing Defensive Medicine	Ilie, A. C., Pîslaru, A. I., Crăcană, I., Ştefăniu, R., & Alexa, I. D	2015			x	x			
Informed consent and Italian physicians: change course or abandon ship—from formal authorization to a culture of sharing	Turillazzi, E., & Neri, M	2015	x	x					
Malpractice lawsuits and change in work in Japanese surgeons	Nakamura, N., & Yamashita, Y	2015		x					
Perspectives and practical applications of medical oncologists on defensive medicine (SYSIPHUS study): a study of the Palliative Care Working Committee of the Turkish Oncology Group (TOG)	Tanriverdi, O., et., al	2015	x						
Consent, Refusal, and Waivers in Patient-Centered Dysphagia Care: Using Law, Ethics, and Evidence to Guide Clinical Practice	Horner, J., Modayil, M., Chapman, L. R., & Dinh, A	2016	x		x	x			
Obstetric and gynecologic malpractice claims in Saudi Arabia: Incidence and cause	AlDakhil, L. O	2016		x					
Choosing Wisely: Law’s Contribution as a Cause of and a Cure for Unwise Healthcare Choices	Ries N. M	2017		x					
Electronic fetal monitoring, cerebral palsy, and medical ethics: Nonsense of a high order	Sartwelle, T. P., Johnston, J. C., & Arda, B	2017	x						
Doctors’ perception of support and the processes involved in complaints investigations and how these relate to welfare and defensive practice: a cross-sectional survey of the UK physicians	Bourne et., al	2017		x					
The determinants of defensive medicine practices in Belgium	Vandersteegen, T., et., al	2017	x						
Second victims in health care: current perspectives	Ozeke, O., Ozeke, V., Coskun, O., & Budakoglu, I. I	2018		x					
A multimodal high-value curriculum affects drivers of utilization and performance on the high-value care component of the internal medicine in-training exam	Chau, T., & Loertscher, L	2018		x				x	
Defensive Medicine and the Imposition of a More Demanding Standard of Care	Vera Lúcia Raposo	2019	x						
Is your mind set? – how are intra- and interpersonal competences dealt with in medical education? A multi-professional qualitative study	Lombardo, L., Ehlers, J., & Lutz, G	2019		x		x	x		
The effects of criminal punishment on medical practices in the medical environment	Lee, M	2019	x	x					
Evidence-based medicine, shared decision making and the hidden curriculum: a qualitative content analysis	Braschi, E., Stacey, D., Légaré, F., Grad, R., & Archibald, D	2020	x						
Risk work in dental practices: an ethnographic study of how risk is managed in NHS dental appointments	Laverty, L., & Harris, R	2020	x						
A colonized general practice? A critical habermasian analysis of how general practitioners experience defensive medicine in their everyday working life	Assing Hvidt, E., Bjørnskov Pedersen, L., Lykkegaard, J., Møller Pedersen, K., & Andersen, M. K	2021	x	x					
How competitors become collaborators—Bridging the gap(s) between machine learning algorithms and clinicians	Grote, T., & Berens, P	2022	x						
A qualitative interview study of Australian physicians on defensive practice and low value care: “it’s easier to talk about our fear of lawyers than to talk about our fear of looking bad in front of each other”	Ries, N. M., Johnston, B., & Jansen, J	2022	x	x			x		

#### Operations and defensive medicine

The role of guidelines is primarily discussed in papers as linked to the practice of operations. In particular, if, on the one hand, guidelines are essential for standardising healthcare activities (indeed, the primary purposes of standardisation are the continuous improvement of quality and the appropriateness and effectiveness of healthcare linked to the reduction of costs), on the other, guidelines are more oriented towards avoiding lawsuits instead of improving quality of care and guaranteeing good practice [7, 42, 59]. These studies declare that guidelines are in favour of defensive medicine. Often, what could have been good practice in the past is not necessarily good in the present [83]. Hence, guidelines, to be effective and reduce defensive medicine, must be flexible and, above all, must be evaluated and used with medical ethics by physicians [8]. For example, Sartwelle et al. [29] find that strictly following the guidelines can cause serious harm to patients since doctors and hospitals are willing to compromise their medical ethics to protect themselves from lawsuits. In other words, using guidelines for self-protection seems to take precedence over medical ethics and the search for the best standard of care.

For these reasons, other studies try to find solutions to the problem of guidelines by underlining the pivotal role played by communications between doctors (for example, the determinant physician’s access to an incident reporting system is found to have a significant impact on most defensive medicine measures [66] or between doctors and patients [43, 47, 54]. Other solutions related to the use of guidelines and the reduction of defensive medicine consist of the optimal use of clinical files (guidelines for good practice as mentioned by Domingues et al. [36], the improvement of the education of trainees and doctors, and gaining a better understanding of the different dynamics of medical disputes [17, 55]. For example, the latter can also happen through improving activities with modern practices [23, 40].

#### Organizising and staffing and defensive medicine

The management of human resources represents one of the most critical functions for healthcare companies. Management must be able to coordinate and combine the various existing professionals (health and non-health) and activate mechanisms, including incentives, to drive the behaviour of individuals to achieve single and company objectives and provide high-quality services. Consequently, organising and staffing are, together with operations, the most observed managerial practice in the sample, being discussed together in several documents [7, 22, 54, 55, 59, 60]. In the documents related to organising and staffing practices, the main problem addressed by the authors is the lack of support from superiors [21, 73] in recognising, dealing with, and learning from errors [57, 62] and as a deterrent to defensive medicine, mainly when residents are considered [78]. In particular, the lack of a healthy team structure with a low hierarchy makes it hard for individuals to deal openly with their uncertainties and mistakes, thus allowing reflection and growth on a personal level [57]. In this sense, it is important to reshape the institution’s medical culture and practice so physicians have a greater awareness of potential costs in their subsequent practice [31]. Finally, the work of Ozeke et al. [76] considers organising and staffing practices only marginally, specifically when the authors talk about how educating doctors about risk may limit defensive medicine and its negative consequences.

#### Monitoring, targets and defensive medicine

The monitoring and target areas focus on measuring and evaluating performance and related mechanisms. Therefore, these practices are closely linked to operations and human resource management as they concern the entire organisation. These practices are also fundamental for guaranteeing a continuous improvement of activities favoured by clear and structured paths for defining problems, feedback systems, corrective actions, and anticipating and managing consequences [48]. Mankaka et al. [62] stated that there is a lack of adequate monitoring regarding the tasks assigned and performed. This lack favours defensive medicine due to the difficulties in recognising and dealing with errors [47].

#### Incentives and defensive medicine

Considering the practice of offering incentives, papers related to incentive mechanisms were considered. Incentives are not only financial and can be linked to individual performance and activities. For example, the culture and practice patterns of training environments are crucial forces in shaping physicians’ cost consciousness, reducing defensive medicine, and improving performance at personal and organisational levels. Education therefore plays a key role in improving doctors’ skills and tools and reducing defensive medicine. Therefore, the institutional culture plays an important role in promoting and incentivising best practices [31, 78].

## Discussion

The objective of this paper was to study the defensive medicine phenomenon from a managerial viewpoint by considering managerial practices [52, 80]. Two databases, WoS and Scopus, were used, with 2011 taken as a start date, and data were selected following the PRISMA guidelines.

In the first part of the analysis, some descriptive data were presented. Starting from these, and in line with [26] it is interesting to stress that quantitative methods dominate many of the studies considered. For this reason, information collected in the literature is not entirely reliable due to the intrinsic limit of surveys, namely their higher subjectivity and the influence of physicians’ beliefs and the organisational environment. Despite a growing interest in defensive medicine worldwide, the phenomenon has been mainly studied in the USA. This result suggests relationships between medicine practices and political-economic-cultural determinants that sometimes work cooperatively and evolve in parallel. The specific cultural (e.g., emotional mechanisms), legal, political, and economic systems of a country can foster (or hinder) the defensive medicine phenomenon. However, although the body of US research may influence – sometimes inaptly – perceptions of legal risks in other countries [26], Germany, Italy, and Switzerland have the highest impact of all countries within the research community.

Moving toward managerial practices, the analysis saw the elimination of many documents unrelated to managerial aspects and the organisational level. This suggests that the defensive medicine phenomenon is more linked to medical areas, even though it can be considered a versatile issue that also concerns economic and managerial considerations. As also suggested by a recent literature review on the definition of defensive medicine in European medical literature, defining defensive medicine is, however, complex, and different interpretations, as well as interesting proposals and possible solutions, can be derived [13].

Indeed, although in the screening phase, the search keys specifically included managerial aspects, in the eligibility phase, 345 studies were eliminated as they are more focused on a single medical specialty (with particular regard to those at high risk of litigation, such as general medicine, surgery, orthopaedic surgery, and obstetrics/gynaecology), without mentioning managerial aspects or the organisation in its purest essence. This is also confirmed by the journal publishing activities analysis (only one paper is published in a journal covering business and management) and by the authors’ affiliation (the predominant area is the medical one).

On the other hand, many contributions focused on physicians, highlighting the different approaches they might take toward their patients or focusing on defensive medicine’s emotional and psychological impacts.

The most recurrent managerial practices are operations and staff organisation. The first is mainly related to the importance and role of guidelines that can limit the repetition of mistakes, particularly when they are strictly connected to an efficient informative system among physicians and between physicians and patients. Nevertheless, if guidelines are not flexible and when the organisation itself lacks a culture able to recognise, monitor, and manage errors, guidelines are unsuccessful, favouring the adoption of defensive behaviours. Thus, another aspect that emerged is the absence of the right incentives and of education which will encourage responsible activity driven by medical ethics rather than fear of lawsuits, as also stated by the recent narrative review of Miziara & Miziara [69]. Indeed, although guidelines which aim to collect (adverse) clinical events in a specific medical area can help prevent such errors, the fear of malpractice litigation is currently too high.

Additionally, analysis of the documents suggests the lack of a culture of disclosure of information, which would identify a higher level of professionalism. Indeed, communications and reports are central aspects of clinical skills because better information leads to less malpractice, whether voluntary or involuntary.

The analysis therefore seems to show the absence of a managerial approach at an organisational level capable of grasping and reducing the problem. Perhaps it is also for this reason that the issue of defensive medicine is not dealt with under the managerial aspect but focuses mainly on the doctor–patient relationship or the macro-system level. Orientation and managerial tools, accompanied by an adequate organisational culture, must be seen as an opportunity to improve the effectiveness and efficiency of health facilities and professionalism of doctors and not, as often happens, a punitive tool. Therefore, and in line with [64], it is important to implement systems to identify some such errors before they cause harm, at either the hospital or the physician level. In other words, it is necessary to stimulate the development of a more profound culture for managing clinical risk which involves administrations and individuals in shared processes and actions. It is needed better to share information and errors, better plan activities, and create a new professional awareness that combines planning-management-reporting of events and activities to encourage the creation of a safety culture within a culture of quality [71] and the development of a new daily practice which is safer for patients and the everyday work of physicians.

### Future research agenda

From a general perspective, this study shows that:

- most analyses in this area were conducted in the USA;
- data were mainly collected through quantitative approaches;
- few studies address defensive medicine from a purely managerial aspect;
- there is an underrepresentation of medical fields;
- studies are mainly oriented toward high-risk medical practices.

Since the results show that this phenomenon has mainly been studied in the USA (and only in a few other countries), it could be interesting to further investigate in depth whether this relationship is the result of interactions between medical practices and political, economic, health insurance, and cultural determinants that sometimes work cooperatively and evolve in parallel, or if other causes contribute to this relationship. It could also be interesting to see if any relationship exists between the defensive medicine and the presence of lawyers in a specific country. Indeed, the presence of lawyers who specialise in medical malpractice cases can encourage healthcare providers to practice defensively. It is no coincidence – maybe – that the concept of defensive medicine was born in the United States, one of the countries with the most significant number of lawyers in the world.

The review identified a gap in the literature, namely the need to improve qualitative research in different countries and fields to enhance the generalisability of results.

Moving to a more specific perspective, defensive medicine from the managerial viewpoint, this review reveals limited interest in these terms, particularly in relation to managerial practices. For this reason, new investigations should highlight the role of managerial instruments as tools to address the phenomenon of defensive medicine according to a risk management system and to move away from reactive management of claims, mainly delegated to insurance companies, to ensure adequate preventive control. For this reason, it is also important to consider culture control within the defensive medicine phenomenon, including managerial culture, planning and budgeting practices, and other managerial control tools – that, in the light of the results of this review, have not been considered – that represent a fundamental basis of the entire managerial mechanism of companies. The proposal for new tools is essential, particularly during a period characterised by the Covid-19 pandemic, which has absorbed not only attention but resources, often to the detriment of the ordinary functionalities of hospital wards (difficulty in managing the structures and in communicating between facilities, departments, colleagues etc.) and has favoured the proliferation of defensive medicine. In particular, it is necessary to find adequate organisational solutions aimed at, on the one hand, reducing the level of litigation and, on the other, guaranteeing a higher level of cost-effectiveness in the healthcare system, particularly under the health service supply.

In the examined studies, the main interlocutors of investigations are physicians, who can transmit non-objective information (affected by a defensive and self-referential approach). It would be appropriate to change the point of view of analysis, considering administrative figures in the investigations to give a different perspective on defensive medicine and different results/answers, to improve the management and organisational aspects as well. In addition, observing managerial practices and their functioning, instead of just the subjects’ behaviour, could lead to more comparable results. Studying managerial practices also favours a more accessible possibility of operationalisation than individual behaviours and traits observable in an individual's actions.

### Limits of the study

This work presents some limitations. First, the work includes only a few studies in its analysis due to the authors’ particular desire to do an initial classification of defensive medicine from a managerial perspective. For this reason, and to achieve higher generalisability of the results, the sample can be enriched by considering other databases to enhance the number of results obtained and different methods of analysis regarding the criteria by which cases are identified. Secondly, only selecting articles written in English may have caused other relevant documents to be omitted.

## Conclusions

This literature review addresses the defensive medicine phenomenon from the managerial perspective. As such, it aims to provide an alternative perspective on this complex phenomenon, which arises from its multifaceted nature encompassing medical, economic, and legal aspects and is influenced by the beliefs and values of a society.

Following some statistical analyses and a study of the managerial practices most frequently addressed in works published from 2011 to June 2022, this work offers a critical analysis of the phenomenon, emphasising the importance of strengthening the managerial aspect and culture to reduce clinical risks, defensive medicine, and enhance healthcare quality, as well as the well-being of both doctors and patients. In doing so, the work paves the way for new research avenues that can improve the quality of future research on defensive medicine.

## Data Availability

NA.

## Footnotes

1 For instance, the work of He A. J. [44] was excluded because it considers the defensive medicine phenomenon from the macro (system) and micro (physician–patient) perspectives. The study sheds fresh light on China’s recent health policy reforms by highlighting the critical impact of the doctor-patient relationship. The Author also suggests that reducing tensions between doctors and patients and reforming the compensation system to allow doctors to respond to the right incentives is necessary to contain healthcare costs.

2 The work of Sartwelle, T. P., Johnston, J. C., & Arda, B. [29] was included in the final analysis because it is closely connected to “operations” managerial practice as it analyses and criticises the standardisation of procedures and use of guidelines used by doctors to protect themselves from lawsuits. Contrariwise, other works – while referring to aspects related to the organisation – were excluded because they did not align with the selected managerial practices. This is the case, for instance, for the study of Chen J. et al. [32], which is oriented at quantifying the monetary impact and radiation exposure related to the radiographic workup of trauma patients.

## References

[1] Abduljawad A, Al-Assaf AF (2011). Incentives for better performance in health care. Sultan Qaboos Univ Med J.

[2] Adwok J, Kearns EH. Defensive medicine: effect on costs, quality and access to healthcare. J Biol Agric Healthc. 2013;3(6):29–35.

[3] AlDakhil LO (2016). Obstetric and gynecologic malpractice claims in Saudi Arabia: Incidence and cause. J Forensic Leg Med.

[4] Antoci A, Maccioni AF, Galeotti M, Russu P (2019). Defensive medicine, liability insurance and malpractice litigation in an evolutionary model. Nonlinear Anal Real World Appl.

[5] Aranaz Andres JM, Valencia-Martín JL, Vicente-Guijarro J, Diaz-Agero Perez C, López-Fresneña N, Carrillo I, SOBRINA Working Group (2020). Low-Value clinical practices: knowledge and beliefs of Spanish surgeons and anesthetists. Int J Environ Res Public Health.

[6] Assing Hvidt E, Bjørnskov Pedersen L, Lykkegaard J, Møller Pedersen K, Andersen MK (2021). A colonized general practice? A critical habermasian analysis of how general practitioners experience defensive medicine in their everyday working life. Health.

[7] Avraham R (2011). Clinical practice guidelines: the warped incentives in the U.S. healthcare system. Am J Law Med.

[8] Baicker K, Fisher ES, Chandra A (2007). Malpractice Liability costs and the practice of medicine in the medicare program. Health Aff.

[9] Baier-Fuentes H, Merigó JM, Amorós JE, Gaviria-Marín M (2019). International entrepreneurship: a bibliometric overview. Int Entrep Manag J.

[10] Barzelay M. The new public management: Improving research and policy dialogue (Vol. 3). Univ of California; 2001.

[11] Baungaard N, Skovvang PL, Hvidt EA, Gerbild H, Andersen MK, Lykkegaard J (2022). How defensive medicine is defined in European medical literature: a systematic review. BMJ Open.

[12] Berlin L (2017). Medical errors, malpractice, and defensive medicine: An ill-fated triad. Diagnosis.

[13] Berwick DM, Hackbarth AD (2012). Eliminating waste in US health care. JAMA.

[14] Bloom N, Genakos C, Sadun R, Van Reenen J (2012). Management practices across firms and countries. Acad Manag Perspect.

[15] Bloom N, Sadun R, Van Reenen J (2012). Does management really work? How three essential practices can address even the most complex global problems. Harv Bus Rev.

[16] Bloom N, Van Reenen J (2007). Measuring and explaining management practices across firms and countries. Q J Econ.

[17] Bouckaert G, Halligan J. Managing performance: International comparisons. New York: Routledge; 2007.

[18] Bourne T, De Cock B, Wynants L, Peters M, Van Audenhove C, Timmerman D, Van Calster B, Jalmbrant M (2017). Doctors’ perception of support and the processes involved in complaints investigations and how these relate to welfare and defensive practice: a cross-sectional survey of the UK physicians. BMJ Open.

[19] Braschi E, Stacey D, Légaré F, Grad R, Archibald D (2020). Evidence-based medicine, shared decision making and the hidden curriculum: a qualitative content analysis. Perspect Med Educ.

[20] Brateanu A, Schramm S, Hu B, Boyer K, Nottingham K, Taksler GB, Jolly S, Goodman K, Misra-Hebert A, Vakharia N, Hamilton AC, Bales R, Manne M, Lathia A, Deshpande A, Rothberg MB (2014). Quantifying the defensive medicine contribution to primary care costs. J Med Econ.

[21] Buelow JR, Winburn K, Hutcherson J (1999). Job satisfaction of home care assistants related to managerial practices. Home Health Care Serv Q.

[22] Burke, J. C., Rosen, author. ), Jef, Minassians, author. ), Henri, Lessard, author. ), Terr, & State Univ. of New York, Albany. N. A. R. Inst. of G. (2000). Performance Funding and Budgeting: An Emerging Merger? : The Fourth Annual Survey (2000) [Article; Article/Report; Microform]. [Washington, D.C.] : Distributed by ERIC Clearinghouse. https://trove.nla.gov.au/work/33300846.

[23] Cartabellotta A (2015). Less is more: un approccio di sistema contro la medicina difensiva. Monitor I Quaderni.

[24] Castillo-Vergara M, Alvarez-Marin A, Placencio-Hidalgo D (2018). A bibliometric analysis of creativity in the field of business economics. J Bus Res.

[25] Chadegani AA, Salehi H, Yunus MM, Farhadi H, Fooladi M, Farhadi M, Ebrahim NA (2013). A comparison between two main academic literature collections: web of science and scopus databases. Asian Soc Sci.

[26] Chau T, Loertscher L (2018). A multimodal high-value curriculum affects drivers of utilization and performance on the high-value care component of the internal medicine in-training exam. J Commun Hosp Internal Med Perspect.

[27] Chen J, Majercik S, Bledsoe J, Connor K, Morris B, Gardner S, Scully C, Wilson E, Dickerson J, White T (2015). The prevalence and impact of defensive medicine in the radiographic workup of the trauma patient: A pilot study. Am J Surg.

[28] Congress U (1994). Office of technology assessment, defensive medicine and medical malpractice.

[29] Di Gregorio, V., Ferriero, A., Specchia, M., Capizzi, S., Damiani, G., & Ricciardi, W. Defensive medicine in Europe: Which solutions? Eur J Public Health, 2015; 25(suppl_3). 10.1093/eurpub/ckv171.043.

[30] Domingues AP, Moura P, Vieira DN (2014). Lessons from a decade of technical–scientific opinions in obstetrical litigation. J Forensic Leg Med.

[31] Dove JT, Brush JE, Chazal RA, Oetgen WJ (2010). Medical Professional Liability and Health Care System Reform. J Am Coll Cardiol.

[32] Dückers M, Faber M, Cruijsberg J, Grol R, Schoonhoven L, Wensing M (2009). Safety and Risk Management Interventions in Hospitals. Med Care Res Rev.

[33] Ezza, A. Le pratiche manageriali delle aziende sanitarie italiane. Un modello per la valutazione. Aracne. 2020. https://books.google.it/books?id=8PjxzQEACAAJ.

[34] Feess E (2012). Malpractice liability, technology choice and negative defensive medicine. Eur J Health Econ.

[35] Gnerre P, Montemurro D, Rivetti C, Palermo C (2016). Choosing wisely: appropriatezza in medicina.

[36] Grote T, Berens P (2022). How competitors become collaborators—Bridging the gap(s) between machine learning algorithms and clinicians. Bioethics.

[37] Hamasaki T, Hagihara A (2015). A comparison of medical litigation filed against obstetrics and gynecology, internal medicine, and surgery departments. BMC Med Ethics.

[38] He AJ (2014). The doctor–patient relationship, defensive medicine and overprescription in Chinese public hospitals: evidence from a cross-sectional survey in Shenzhen city. Soc Sci Med.

[39] Hood C (2004). The middle aging of new public management: into the age of paradox?. J Public Adm Res Theory.

[40] Horner J, Modayil M, Chapman LR, Dinh A (2016). Consent, refusal, and waivers in patient-centered dysphagia care: using law, ethics, and evidence to guide clinical practice. Am J Speech Lang Pathol.

[41] Ilie AC, Pislaru AI, Cracana I, Stefaniu R, Dana Alexa I (2015). Home blood pressure monitoring—One step towards reducing defensive medicine. E-Health Bioeng Conference (EHB).

[42] Johansen M, Zhu L (2014). Market competition, political constraint, and managerial practice in public, nonprofit, and private American Hospitals. J Public Adm Res Theory.

[43] Kapp MB (2017). Defensive medicine: no wonder policymakers are confused. Int J Risk Saf Med.

[44] Katz ED (2019). Defensive medicine: a case and review of its status and possible solutions. Clin Pract Cases Emerg Med.

[45] Kotter JP. Leading change. Boston: Harvard business press; 2012.

[46] Laverty L, Harris R (2020). Risk work in dental practices: an ethnographic study of how risk is managed in NHS dental appointments. Sociol Health Illn.

[47] Lee M (2019). The effects of criminal punishment on medical practices in the medical environment. Int J Environ Res Public Health.

[48] Lefton R (2008). Addressing roadblocks to hospital-physician alignment: The Hippocratic Oath is a guiding force behind every decision that a physician makes. But it would be idealistic not to acknowledge that physicians have economic as well as humanitarian interests at stake whenever they take on a new patient or order a diagnostic test. The same, of course, is true of hospitals. Healthc Financial Manag.

[49] Lombardo L, Ehlers J, Lutz G (2019). Is your mind set? – How are intra- and interpersonal competences dealt with in medical education? A multi-professional qualitative study. BMC Med Educ.

[50] Macdonald H, Loder E. Too much medicine: the challenge of finding common ground. BMJ. 2015;350:h1163. 10.1136/bmj.h1163 25739770

[51] Malach M, Baumol WJ (2012). Opportunities for cost reduction of medical care: part 3. J Community Health.

[52] Mankaka CO, Waeber G, Gachoud D (2014). Female residents experiencing medical errors in general internal medicine: a qualitative study. BMC Med Educ.

[53] Mareiniss DP (2015). Waxman et al in NEJM does not disprove defensive practices in the ED. Am J Emerg Med.

[54] Medar C, Cristache CM, Mihut T, Marcov EC, Furtunescu FL, Burlibasa M, Burlibasa L (2020). Defensive dentistry from normal medical practice to safeguard from malpractice litigations. New rules in COVID-19 pandemic. Rom J Leg Med.

[55] Mello MM, Chandra A, Gawande AA, Studdert DM (2010). National costs of the medical liability system. Health Aff.

[56] Mello MM, Frakes MD, Blumenkranz E, Studdert DM (2020). Malpractice liability and health care quality: a review. JAMA.

[57] Mira JJ, Carrillo I, Pérez-Pérez P, Olivera G, Silvestre C, Nebot C, Aranaz-Andrés J (2018). Level of knowledge of Quality Commitment Campaign and of" do not do" recommendations amongst general practitioners, pediatricians and nurses Primary Care. An Del Sistema Sanitario De Navarra.

[58] Miziara ID, Miziara CSMG (2022). Medical errors, medical negligence and defensive medicine: A narrative review. Clinics.

[59] Moher D, Liberati A, Tetzlaff J, Altman D. G, The PRISMA Group. Preferred Reporting Items for Systematic Reviews and Meta-Analyses: The PRISMA Statement. PLoS Med. 2009;6(7):e1000097. 10.1371/journal.pmed.1000097 PMC2707599 19621072

[60] Morris JA, Carrillo Y, Jenkins JM, Smith PW, Bledsoe S, Pichert J, White A (2003). Surgical adverse events, risk management, and malpractice outcome: Morbidity and mortality review is not enough. Ann Surg.

[61] Moynihan R, Glasziou P, Woloshin S, Schwartz L, Santa J, Godlee F. Winding back the harms of too much medicine. BMJ. 2013;346:f1271. 10.1136/bmj.f1271 23444422

[62] Nakamura N, Yamashita Y (2015). Malpractice lawsuits and change in work in Japanese surgeons. J Surg Res.

[63] O’Connell J (2021). Defensive pharmacy practice: a gap in our understanding. Int J Clin Pharm.

[64] O'Leary KJ, Choi J, Watson K, Williams MV. Medical students' and residents' clinical and educational experiences with defensive medicine. Acad Med. 2012;87(2):142–8. 10.1097/ACM.0b013e31823f2c86. 10.1097/ACM.0b013e31823f2c86 22189882

[65] Ozeke O, Ozeke V, Coskun O, Budakoglu II (2019). Second victims in health care: current perspectives. Adv Med Educ Pract.

[66] Pathirana T, Clark J, Moynihan R. Mapping the drivers of overdiagnosis to potential solutions. BMJ. 2017;j3879. 10.1136/bmj.j3879. 10.1136/bmj.j3879 28814436

[67] Paul J, Criado AR. The art of writing literature review: What do we know and what do we need to know?. Int Bus Rev. 2020;29(4):101717.

[68] Pellino IM, Pellino G (2015). Consequences of defensive medicine, second victims, and clinical-judicial syndrome on surgeons’ medical practice and on health service. Updat Surg.

[69] Pines JM, Farmer SA, Akman JS (2014). “Innovation” institutes in academic health centers: enhancing value through leadership, education, engagement, and scholarship. Acad Med.

[70] Raposo VL (2019). Defensive medicine and the imposition of a more demanding standard of care. J Leg Med.

[71] Ries NM (2017). Choosing wisely: law’s contribution as a cause of and a cure for unwise healthcare choices. SSRN Electron J.

[72] Ries NM, Jansen J (2021). Physicians’ views and experiences of defensive medicine: an international review of empirical research. Health Policy.

[73] Ries NM, Johnston B, Jansen J (2022). A qualitative interview study of Australian physicians on defensive practice and low value care:“it’s easier to talk about our fear of lawyers than to talk about our fear of looking bad in front of each other”. BMC Med Ethics.

[74] Sartwelle TP, Johnston JC, Arda B (2017). Electronic fetal monitoring, cerebral palsy, and medical ethics: Nonsense of a high order ^1^. Med Law Int.

[75] Schneider A (2019). Defensive medicine practice and effect on healthcare expenditures and tort reform. Nurse Care Open Access J.

[76] Studdert DM (2005). Defensive Medicine Among High-Risk Specialist Physicians in a Volatile Malpractice Environment. JAMA.

[77] Symon A. Obstetric Litigation: Effects on Clinical Practice^1^. Gynäkologisch-Geburtshilfliche Rundschau. 2000;40(3–4):165–71. 10.1159/000053021. 10.1159/000053021 11326163

[78] Syverson C (2004). Product substitutability and productivity dispersion. Rev Econ Stat.

[79] Tanriverdi O, Cay-Senler F, Yavuzsen T, Turhal S, Akman T, Komurcu S, Cehreli R, Ozyilkan O (2015). Perspectives and practical applications of medical oncologists on defensive medicine (SYSIPHUS study): A study of the Palliative Care Working Committee of the Turkish Oncology Group (TOG). Med Oncol.

[80] Turillazzi E, Neri M (2015). Informed consent and Italian physicians: Change course or abandon ship—from formal authorization to a culture of sharing. Med Health Care Philos.

[81] Vandersteegen T, Marneffe W, Cleemput I, Vandijck D, Vereeck L (2017). The determinants of defensive medicine practices in Belgium. Health Econ Policy Law.

[82] Vento S, Cainelli F, Vallone A (2018). Defensive medicine: It is time to finally slow down an epidemic. World J Clin Cases.

[83] Williams PL, Williams JP, Williams BR (2021). The fine line of defensive medicine. J Forensic Leg Med.

